# Prebiotics Do Not Influence the Severity of Atopic Dermatitis in Infants: A Randomised Controlled Trial

**DOI:** 10.1371/journal.pone.0142897

**Published:** 2015-11-16

**Authors:** Jan Boženský, Martin Hill, Richard Zelenka, Tomáš Skýba

**Affiliations:** 1 Paediatrics Department, Vitkovice Hospital, Ostrava, Czech Republic; 2 Institute of Endocrinology, Prague, Czech Republic; 3 DMK Baby, Bremen, Germany; Penang Medical College, MALAYSIA

## Abstract

The objective was to evaluate the effects of a hypoallergenic (HA) formula supplemented with prebiotic galacto-oligosaccharides on the severity of atopic manifestations. A randomised clinical trial was conducted. The control group was infants, fed with hypoallergenic formula and without supplementation. The duration of the study was six months. The primary outcome of the study was a difference in the severity of atopic dermatitis measured using SCORAD (Scoring Atopic Dermatitis) criteria. Secondary outcomes were anthropometry (length, weight, and head circumference), together with the tolerance and incidence of infections. Both groups showed a decrease of average SCORAD values, but no statistically significant difference between the evaluated groups was observed. There were no statistically significant differences in anthropometry, or the tolerance or incidence of infections. Although there is no evidence, that consumption of a hypoallergenic infant formula enriched with prebiotic galacto-oligosaccharides had any effect on SCORAD, it was safe and well tolerated.

**Trial Registration:**

www.clinicaltrials.gov NCT 02077088

## Introduction

Breastfed infants have a significantly higher concentration of bifidobacteria in stools and a lower complexity of stool microflora than their artificially fed counterparts. With age and the introduction of complementary foods, these differences gradually disappear [1]. One of the bifidogenic factors present in breast milk are oligosaccharides [2, 3]. One such oligosaccharide group, galacto-oligosaccharides (GOS) is used as a functional ingredient of infant formula. Their bifidogenicity and safety in infant formulas are well documented [4]. The use of GOS in formulas is accompanied by softer stool consistency.

A major issue in paediatrics is a rising prevalence of allergy in general and of food-related allergies, such as cows’ milk protein allergy (CPMA) in particular. It can be stated that in about one third of children with atopic eczema the underlying cause is cows’ milk protein allergy [5]. Cows’ milk protein allergy can have a wide variety of symptoms, such as vomiting and diarrhoea [6, 7]. Nevertheless, atopic dermatitis remains one of the most important ones. Based upon an analysis of available evidence the European Academy of Allergy Studies and Clinical Immunology recommends breastfeeding as the most effective measure in preventing CPMA [8].

The preventive effect of infant formula containing partially hydrolysed cows’ milk protein has been observed in several clinical studies [9, 10]. An increased preventive effect of such formula due to addition of a combination of 90% galacto-oligosaccharides and 10% fructo-oligosaccharides has been described [11, 12, 13]. The existence of a causality was later refuted by the European Food Safety Agency /EFSA/ [14] due to issues in study design and execution. Therefore it became relevant to study to what extent similar results related to the preventive effect of prebiotic oligosaccharides on dermal symptoms of infant atopy could be observed when using 100% galacto-oligosaccharides as the prebiotic source. SCORAD was chosen as an objective measure of the extent of atopy.

## Methods

### Study Design

A randomised control study was performed in collaboration with paediatricians in the Ostrava region (Czech Republic). We included full-term infants with a positive history of allergy in their parents or siblings (atopic eczema, allergic rhinitis and/or asthma). Their age at recruitment was 6–8 weeks. We excluded breast-fed children, children on some other formula, and those with gastrointestinal disorders. At inclusion the infants were fed with hypoallergenic formula. Hypoallergenic study and control formulas were provided by HUMANA, Germany.

A prerequisite for inclusion into the study was the termination of breastfeeding before 6 weeks of life and informed consent signed by both parents. The study protocol submitted in 2 language versions (**S1 File** and **S2 File**) was authorised by the responsible ethics committee at Ostrava-Vitkovice Hospital. All clinical investigations were conducted according to principles set out in the Helsinki Declaration.

Sample size was calculated based on statistical power analysis. This was based on the primary outcome, SCORAD values at six months of age, assumptions about their differences and variations in standard deviation are based on published data [15]. Magnitude of differences between SCORAD scores of the groups was assumed to be similar the one observed in [15] when analysing incidence of atopy at 6 months of age. At a power of 0.8 and a significance level of alpha = .05, it was concluded that 98 children would be necessary for the study The infants were randomised into two groups (based on a computer-generated randomisation list). In the first group they received formula with hydrolysed protein (HA); the second group received an identical formula with a supplement of 0.5g/100 ml of galacto-oligosaccharides (GOS). The composition of formulas complied with the provisions of valid European legislation. Randomisation was performed by an independent consultant, and decoding was only done after data analysis. Participants were enrolled by family paediatricians, assignment by one of the authors (TS). During the study, infants were excluded due to violations of the study protocol, non-compliance, and/or illness.

### Study Outcomes

The primary hypothesis was the existence of a statistically significant difference in intensity of atopic manifestations (SCORAD). The primary outcome measured was the difference in average SCORAD values at three and six months of age between control and supplemented groups. SCORAD (Scoring Atopic Dermatitis) is a clinical tool widely used to asses the extent and severity of eczema. Secondary outcomes were average weight, length and head circumference, stool frequency and consistency, vomiting (gastro-oesophageal reflux), and the incidence of infections and possible adverse effects.

Infants were examined by a paediatrician at inclusion and then at three and six months of age. The examination included anthropometry (height, weight, and head circumference) and the presence of atopic dermatitis based on SCORAD criteria. Calculation was performed using a corresponding software application (http://adserver.sante.univ-nantes.fr). To ensure consistency, all examinations were performed in the same clinical ward alternatively by two paediatricians. Body weight was measured using SECA scales (accuracy ±25g). Length and head circumference were measured by standard methods. Other parameters such as the extent of vomiting and colic episodes were noted by parents in a questionnaire. These data were noted on a daily basis, transformed into an Excel file and used for statistical evaluation. The study was originally registered under EUDRACT 2008-005488-32. Because this was not a WHO approved database, the study was re-registered at www.clinicaltrials.gov under NCT02077088. The authors confirm that all on-going and related trials for this intervention have been registered.

### Statistical Methods

The relationships between SCORAD and gender differences, the effects of prebiotics and age of infants were simultaneously evaluated using a repeated measures ANOVA model, Friedman and chi-squared tests. The software used was the Statgraphics Centurion XVI package (Statpoint, Herndon, USA).

Variables showing non-symmetric data distribution and non-constant variance were transformed by a power transformation prior to statistical testing to attain distributional symmetry and constant variance [16]. Non-homogeneities in the transformed data were detected using residual analysis as described elsewhere [17]. The ANOVA model was followed by least significant difference multiple comparisons. The optimum transformation parameter was found at maximum correlation between actual percentiles and Gauss-distribution percentiles. Nominal data about stool frequency, distribution etc. were analysed using the chi-squared test. Results were analyzed on a per protocol basis.

## Results

### Subjects

**Fig 1:**
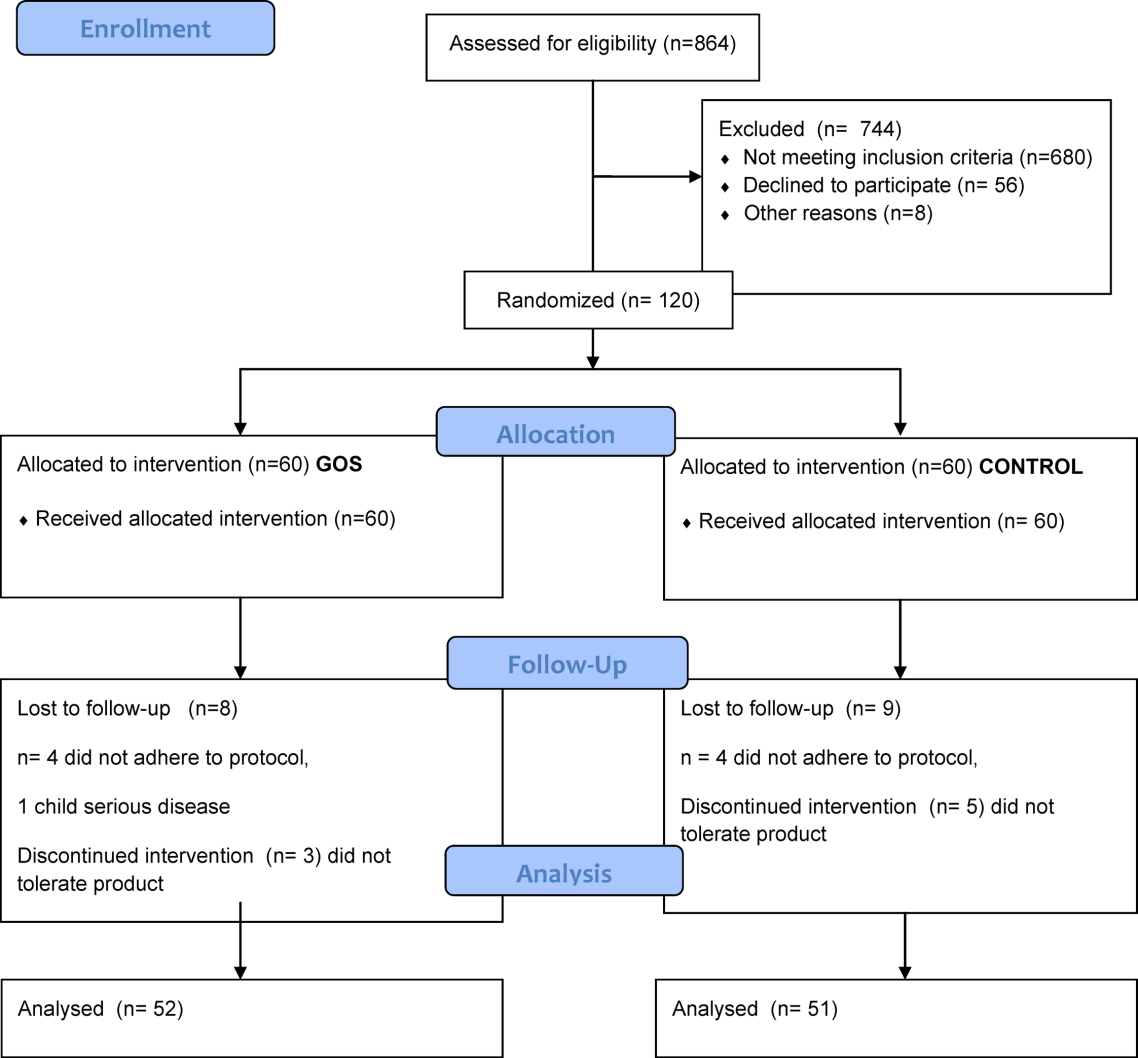
Flow-chart of infant participation (CONSORT 2010) Corresponding checklist is provided as S1 CONSORT Checklist.

In total, 120 infants (67 boys and 43 girls) were included in the study, and 17 dropped out. The most frequent reason was non-compliance (8) and protocol violation (6). Only two infants did not tolerate the formula; one child was excluded due to other serious illness. Recruitment and data collection were performed between October 2008 and May 2012. Details about study flow can be found in the corresponding CONSORT 2010 flowchart [18] (Fig 1). Table 1 shows demographic characteristics of the infants; no significant differences were observed between the two groups.

**Table 1:** Demographic characteristics of the study and control groups. Study n = 52, Control n = 51.

	Control (HA)	Study (HA+GOS)
Vaginal birth (%)	72.1	73.4
Age of mother at birth (years ±sd)	29.3 ±3.2	31.5 ±3.1
Mother an active smoker (%)	17	15
Pet in family (%)	13	16

### Evaluation of atopic symptoms–SCORAD

**Fig 2:**
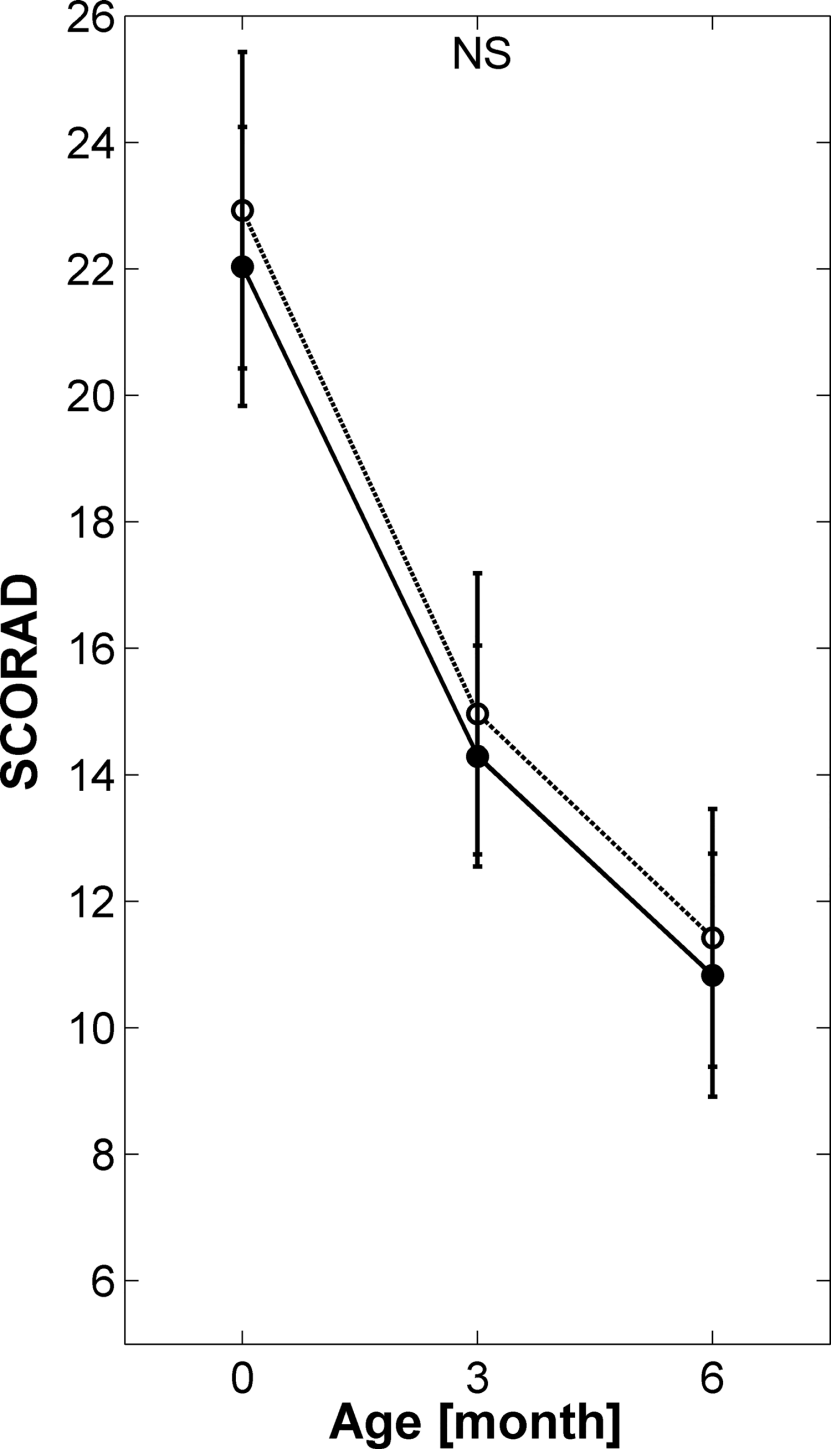
Comparison of SCORAD score. Bars represent 95% CI intervals, closed circles study group, open circles control group. At baseline 120 infants were included, at 3 month follow-up 109 infants were included, at 6 months 103 infants finished the study.

Initial SCORAD values decreased gradually over time as can be seen in Fig 2. A comparison between the two groups showed only non-significant differences.

### Stool frequency, fussiness and abdominal colics, vomiting and fever

The results show a reduction in the frequency of episodes of colic or gastro-oesophageal reflux at three and six months of age but no statistically significant difference between the two groups. The frequency of infections (increased body temperature above 37°C) showed no differences over time or between the two groups. Results on this, and data about the effect on stool frequency and consistency can be found in Table 2. Only the effects on stool consistency reached significance. We found no effect of the presence of older siblings in the family on the incidence of infections.

**Table 2:** The influence of prebiotics on clinical data (Study n = 52, Control n = 51). Frequency of stools (1 = no, 2 = 1–4 times, 3 = 5 and more), stool consistency (1 = watery, 2 = runny, 3 = mushy, 4 = formed, 5 = solid), vomiting frequency (1 = no, 2 = 1–4 times, 3 = 5 and more), frequency of crying (1 = no, 2 = 1–3 times, 3 = 4 and more), ns = non-significant.

	Control	Study		
Stool frequency	2.15	2.89	P = 0.52	NS
Stool consistency	3.52	2.78	P<0.05	Significance
Vomiting frequency	2.72	2.15	P = 0.44	NS
Crying frequency	2.36	2.18	P = 0.27	NS

### Anthropometry

Regarding body weight, length, and head circumference, statistical analysis did not show significant differences.

## Discussion

In the study no effect was found of galacto-oligosaccharide-supplemented infant formula on the severity of atopic dermatitis in infants with a positive family anamnesis of allergy.

Breast milk contains high concentrations of structurally complex prebiotic oligosaccharides, predominantly galacto-oligosaccharides. Cows’ milk-derived galacto-oligosaccharides structurally similar to human milk oligosaccharides have in the meantime become a component of commercial infant formula. They were studied mostly using a ratio of 90/10 in combination with plant-based fructo-oligosacharides (FOS). This prebiotic combination showed similar microflora, stool consistency, and pH to breast-fed children [19, 20]. Other studies with this GOS/FOS combination showed a preventive effect in cases of infectious disease and atopic eczema [21]. There are no studies proving such effects with pure galacto-oligosaccharides.

Although Moro et al. [12] observed a decreased incidence of atopy with the GOS/FOS combination, they found no significant effect on SCORAD values. They measured SCORAD values only at 3 and 6 months of age, using standard SCORAD methodology [22]. We decided to analyse to what extent this might be different using pure galacto-oligosaccharides as the prebiotic source.

We observed no significant difference between SCORAD values of the two groups; the only difference observed was a tendency towards a lower six-month SCORAD in boys in the study group. Gender-specific differences were also observed via other parameters such as crying or gastro-oesophageal reflux. To our knowledge, there are no data available which might explain this phenomenon.

Our results seem to confirm a general lack of efficacy of prebiotic fibre in improving atopic dermatitis symptoms. Because the above-mentioned effect on atopy incidence observed by Moro et al. [12] was also questioned by an EFSA analysis, the possible effects of galacto-oligosaccharides and fructo-oligosaccharides on atopy, whether alone or in combination, seem to lack a basis of reliable evidence.

No adverse effects related to the consumption of both formulas were observed. On the contrary, they were well tolerated. Product-related dropout rates were in line with data observed for this age category (for details, see Results section), and there were no differences between the groups. Feeding-related behaviour showed no significant differences between the groups. A borderline significance of the effects of GOS on stool consistency was observed, with the experimental group having slightly softer stools.

Consistent with the observed lack of efficacy of added galacto-oligosaccharides on atopic dermatitis prevention we could neither observe preventive effects against infections nor an effect on antibiotic prescription.

In summary, we were unable to demonstrate any benefit on atopic dermatitis due to supplementing hypoallergenic formula with prebiotic galacto-oligosaccharides, with possible exception of stool consistency and the prevention of constipation. We found no adverse effects in either product, and have shown safety and excellent tolerance of the formula used. To what extent prebiotics and other functional supplements to infant formula may have an effect on infant health remains to be ultimately decided in future studies, but currently such a relationship seems to be rather improbable.

## Supporting Information

S1 CONSORT Checklist CONSORT 2010 Checklist. (DOC)

S1 File Study Protocol (English). (DOC)

S2 File Study Protocol (Czech). (DOC)
