# Supplementary material for: Prebiotics Do Not Influence the Severity of Atopic Dermatitis in Infants: A Randomised Controlled Trial
Source: PLoS One. 2015 Nov 16;10(11):e0142897. doi: 10.1371/journal.pone.0142897 (PMC4646669; doi:10.1371/journal.pone.0142897)
Supplement: S1 File — (DOC) [file pone.0142897.s002.doc]

Double‑blind, randomised study on the effect of prebiotics

on the incidence of atopic manifestations of infants

## Project no.: 1.090

Date:15.1.2007

**Coordinating Investigator:**

Prim MUDr. Jan Bozensky

**Address: Vitkovicka nemocnice Zaluzanskeho 403, Ostrava**

:

**Sponsor:**

HUMANA GmbH

Bielefelder Str. 66

D-32051 Herford / Germany

Tel: ++49-5221-181-0

Fax: ++49-5221-181-0

###

**2.** PROTOCOL SYNOPSIS

**TITLE:**

**Double‑blind, randomised study on the effect of prebiotics**

**on the incidence of atopic manifestations in infants**

**SPONSOR:**

**HUMANA GmbH**

**Bielefelder Str. 66**

**D-32051 Herford / Germany**

**OBJECTIVES:**

Evaluate the effects of the dietary regimen in infants with a positive family history of atopy on the incidence of atopic complications during the first 6 months of age

**STUDY DESIGN:**

Double‑blind, randomised, controlled prospective study

**PLANNED SAMPLE SIZE:**

60 infants per group, 2 groups,

**SUBJECT SELECTION CRITERIA:**

Infants with a postive family anamnesis of atopy (father or mother or both have a history of atopic dermatitis, allergic rhinitis or asthma) , born during week 37 and 42 of gestation, nonhypotrofic, supplemental feeding with formula will be started no later then 14 days after birth, after 6 weeks of age formula shall be the sole source of nutrtion for the child

**TEST GROUPS:**

2 groups receiving 2 different HA formulas: (standard and enriched with prebiotics).

**Feeding Regimen:**

Ad libitum feeding based needs of the infant

**MAIN PARAMETERS OF EFFICACY:**

Quantitative enumeration of the incidence of atopic symptoms based on SCORAD criteria, evaluation of parental data about incidence of crying, regurgitation, vomiting and stool consistency

**MAIN PARAMETERS OF SAFETY:**

Normal development of growth (length and weight) according to growth charts.

**PROCEDURES:**

**The children will be evalauted three times by a physician (at onset of feeding, after 3 months and after 6 months of age). Other parameters will be monitored by the parents on the basis of a daily diary.**

**STATISTICAL ANALYSIS:**

Statistical analysis of the atopic manifestation incidence will be analyzed separately using Mann-Whitney statistics. Statistical evalu­a­tion of frequen­cies will be performed using a chi-square analysis3. Background Information

It is generally accepted that the colonic microflora is important to health, especially during infancy. There is some evidence that the microflora of breast-fed infants is dominated by populations of bifidobacteria (1). There are many factors discussed to be important for the development of the intestinal ecosystem, some postulations attribute this influence to the feeding regime. Growth factors for bifidobacteria (bifidus factors) present in human milk, mostly oligosaccharides (beside other factors), are the ingredients of human milk which were discussed to be responsible as specific factors for the growth of bifidobacteria (2). Only recently in Europe oligosaccharides were added to a few infant formulas, whereas most hypoallergenic formulas do not contain any oligosaccharides or prebiotics; this may account for the possible differences in the bacterial population found between breast-fed and formula-fed infants. This has led to the consideration that dietary supplements may influence the flora composition. Different approaches are feasible: the use of prebiotics, probiotics or synbiotics (15).

Prebiotics are nondigestible food ingredients that beneficially affect the host by selectively stimulating the growth and/or activity of one or a limited number of bacterial species already resident in the colon (3).

Probiotics are live microbial food supplements that beneficially affect the host by improving its intestinal microbial balance (4).

Synbiotics are the combination of prebiotics and probiotics(5).

Oligosaccharides can be seen as prebiotics, when they are not hydrolysed nor absorbed in the upper gastrointestinal tract and when they stimulate the growth of lactic acid-producing bacteria like lactobacilli or bifidobacteria. Beside a number of different ingredients, two types of oligosaccharide have been studied mostly: inulin/fructo-oligosaccharides and galacto-oligosaccharides.

For this study galacto-oligosaccharides (GOS) have been selected. These oligosaccharides are present naturally in human and cow’s milk and they are found in large amounts in fermented milk products like yoghurt. They are normal products when lactose is hydrolysed by the enzyme β-galactosidase. They are produced in large amounts (15 000 t/y in Japan, 1995) and used since some years in Japan as prebiotics in infant formulas (6). They are used as prebiotic supplements in yoghurt since 1995 in Europe. GOS have a generally recognized as safe (GRAS) status and have shown no toxicity when tested in rats (acute and chronic). GOS are a mixture of different oligosaccharides, whereby the main products are tri- to hexa-saccharides, some lactose, galactose and glucose are also found in the GOS-mixture.

GOS have been tested in humans and have been bifidogenic at daily intakes of 2.5 and 5 g (6) and in another study at 10g per day (7). GOS have been shown to improve calcium absorption in animal studies like it has been shown for other prebiotics (17,18), whereas this was not seen in a human study (6). Other studies in humans have shown that GOS could normalize the stool frequency in constipation (6,8). Since the year 2000 GOS are also used in an infant formulae in Europe.

Simutalneusly it is widely accepted, that in children with a positive allergic anamnesis hypoallergenic infant nutrtion can reduce the inicidence of atopic symptoms or at least delay their onset.(GINI). It became thus interesting to know, if manipulation of the microflora could also have a positive influence on the incidence of atopic disease similar to breasfeeding (2,3), A current paper (4) seems to have found a reduction in incidence of atopic dermatitis after prebiotic supplementation, so it becomes of paramount importance to establish if this phenomenon is limited only to this specific prebiotic mixture or if it is more general.

4. Aim of the study

The aim of the study is to investigate whether the supplementation of a hypoallergenic infant formula with prebiotics can influence primarily the incidence of atopic disease, secondarily wellbeing of the infants and stool characteristics

5. Working hypothesis

It is hypothesised that incidence of atopic dermatitis by infants fed a HA formula supplemented with prebiotics will be at least 30% less than without the sup­plementation.

There will be no differences in wellbeing between the two study groups, stool consistency will be softer in the group with prebiotic supplementation.

6. Control parameters

**Primary parameters:**

Atopic dermatitis incidence and severity evaluated by SCORAD

**Secondary parameters:**

Weight, length , mean daily volume of formula intake and energy intake (calculated).

Stool frequency (number of stools per day),

Stool consistency (according to a standardized score derived from pictures),

Diaper rash (according to a standardized score, registered as sum scores),

Registration of diarrhoeal episodes (according to a diarrhoeal score) and

fever episodes (all temperatures above 37 ^o^C (on the skin) or 37,5^o^C (in the anus) will be considered as fever).

Periods of crying, cramps and regurgitation

**Safety parameters:**

Normal development of growth (length and weight) according to growth charts.

7. Study design

The study will be performed as double blind randomised prospective study .

Infants who will enter the study will be randomly assigned to one of the two formula groups (coded YELLOW and GREEN).

At study day 1 (Onset of fomula feeding) the child will be evaluated by a physician who will determine ist length, weight, head circumference and atopic dermatitis status.

The same will be repeated at study day 2(at 3 months of age) and at study day 3 (6 months of age).

In the meantime the mothers will evalaute the other parameters based on a daily diary like protocol.

**8. Time**

The study will last for 12 months. It will start in March 2007 and the clinical part will be finished presumably in March 2008.

**9. Subjects**

**Healthy nonhypotrofic** infants with a positive allergic family analysis and born at a gestational age between 37-42 weeks.

**9.1 Number**

120 infants per group (60 infants for each group fulfilling the exclusion and inclusion criteria) will be studied.

**9.2 Inclusion criteria**

.

- Healthy nonhypotrofic infants with a positive allergic family analysis and born at a gestational age between 37-42 weeks
- Age at study entry 14 days maximally
- Weight and length appropriate for the respective age based growth charts.
- As supplemental formula feeding to breastfeeding untill 6 weeks of age, then sole source of nutrition untill study end, beikost introduction possible, but should be consulted with physician.
- Written informed consent of parent/guardian

**9.3 Exclusion criteria**

- Exclusively breast fed infants
- Supplemental breastfeeding after 6 weeks of age
- Severe medical problems
- Infant not receiving the group specific formula as the only formula fed
- Any major congenital malformations of the digestive tract
- HIV positive infant (status of the mother is important: testing is only needed for HIV-positive mothers)
- Evidence of significant gastrointestinal diseases
- Participation in any other clinical trial

**10. Study Proceeding**

10.1 Enrolment of patients

Infants fulfilling the requirements of inclusion but not exclusion criteria as stated under para 9.2 and 9.3 and whose parents have been informed about the study and have given their written consent for participation of their child in the study shall be enrolled.

The allocation to one of the two supplementation regimen (prebiotics or placebo) will be carried out randomly according to the time‑balanced randomisation table (Annex I) Randomisation is conducted by use of a computer created randomization table.

Each infant will get an identification code:

nnnn.p (e.g.: 1004.3) where :

nnnn= randomized number allocation

p= study day (1,2)

Additionally the following indices will be taken in the CRFs:

initials (like: H.L.)

day of birth (like: 22.11.2001)

sex: male / female

formula group (YELLOW or GREEN)

10.2 Feeding regimen

2 groups; receiving 2 different dissolved, powdered hypoallergenic formulas: with the following supplementation regimen (prebiotics or standard). The prebiotic hypoallergenic formula contains galacto-oligosaccharides . The standard formula is the basic hypoallergenic formula composed in the similar way like the product HUMANA HA 1, but without any addition. These formulas are coded YELLOW or GREEN.

Feeding will be ad libitum, Formula feeding should start by the age of 14 days, supplementary breastfeeding is allowed until 6 weeks of life.

10.3 Package/label

The formulas will be packaged in powdered form and will be labelled as hypoallergenic formula (colour coded in YELLOW or GREEN). The code for the formulas will be disclosed at the end of the study..

10.6 Statistical analyses

All parameters will be analyzed separately using Mann-Whitney comparisons by rank.

Statistical evalu­a­tion of frequen­cies will be performed using a chi-square analysis. The effect of diet on a group of parameters (i.e. growth parameters, stool plus diarrhoeal parameters) will be tested by multivariate statistics.

10.7 Data collecting

Control parameters as specified under paragraph 6 shall be collected for each subject on each study day.

10.8 Documentation

Case report forms shall be used for recording all findings rele­vant for assessment of the selected parameters (parallel documentation of hospital's patient records).

For each form on which information is entered, the infant's initials, an identification code including the randomisation number, date of birth, sex, and feeding group shall be entered in the appropriate space (details under 10.1).

Errors must be corrected by drawing a single line through the incorrect entry and writing in the new value positioned as close to the original as possible. The correc­tion must then be initialled and dated by the authorized individual making the change In no case obliterate, write over, or erase the original entry when making a correc­tion.

As soon as possible after the end of each infant's participation in the study, the CRFs shall be completed and signed by the principle investigator. The originals will be handed over to the project monitor for review of completeness. Copies of the CRFs may remain in the clinic under lock and key.

10.9 Clinical monitoring

The principal investigators are in charge with guaranteeing continuous monitoring by su­pervising research nurses and physicians he/she has authorized. All findings re­corded during clinical monitoring will be retained for at least 1 year (after finishing the study and the report) and made avail­able upon request of a professional HUMANA representative during a monitoring visit to obtain clinical background information for data evaluation.

10.10 Withdrawals and dropouts

If any subject fails to complete the study (withdrawal from the study, e.g. in case of revocation of parental consent, or dropout, e.g. in case of continued breastfeeding after 6 weeks of age) the reason should be specified in writing by the supervising physician. If parents decide to withdraw her baby from the study without giving any reason this should also be stated.

In case of >30% missing data of an infant the concerned subject will be excluded from the further study and subsequent statistical data evaluation (dropout because of missing data). The same is true if the specific feeding regimen for an individual is discontinued before the study end (dropout because of violation of feeding regimen). Incidental feeding of small amounts of food different from the prescribed feeding regimen will not lead to exclusion.

After having reached the last randomization number in each group any non­completer of the study ‑no matter if withdrawal or dropout‑ will be replaced according to his randomization number. This means that the randomization numbers of non­completers have to be assigned once again. For identification of replaced non­completers the respective randomization numbers should be labelled with the sup­plement "b" (in case of a third allocation of a randomization number with the supple­ment "c" etc.).

For identification of non‑completers as possible non‑responders, the protocols (CRFs) of those cases should be handed over to the local monitor, too. The maxi­mum rate for replacement of study cases is 30%; this limit must not be exceeded, otherwise the validity of the results of the biometrical data evaluation would be ques­tionable.

**________________________________**

^1^ If infants fall ill during the study period, the co-ordinating investigator will decide whether the disease may justify with­drawal of the infant from the study. This will also depend on the type and dose of drug(s) provided for treat­ment.

11. Personal responsibilities

11.1 Responsibility of the participating clinicians

All study related effects reported by the physician, dietician, nurse or by the parents will be recorded and the local supervising physicians will pass details of all untoward effects to HUMANA. The nature of each adverse experience, time, and relationship to the feeding should be established. Those of serious nature and those thought to be associated with the experimental feeding will be reported to HUMANA within 24 hours (Dr. Richard Zelenka, HUMANA, Tel: 00420-731455278, Fax: 0049-5221-181-486 and/or Dr. G. Sawatzki, SciNuTec GmbH, Tel: 0049-6033-73020, Fax: 0049-6033-73024) The study can be inter­rupted by the clinicians at any time if any unforeseen problems arise. HUMANA will be informed within 24 hours about any intention to discontinue or terminate the study before final tests are taken.

11.2 Responsibility of HUMANA

HUMANA will be responsible for the production of all test formulas. Humana will take care that all costs for the study in the clinics will be covered by Humana and no additional expenses will appear.

All data transmitted to HUMANA will be stored adequately.

12. Insurance

HUMANA has a general liability insurance, which covers compensation to all subjects of this study for trial-related injury. The insurance company is Gothaer Versicherungsbank VvaG, Niederlassung Dortmund, Westfalendamm96, Germany and the policy no. is 26.073.558742.

13. Protocol changes

Any proposed changes in the above design will be subject of a written protocol amendment before implementation which will be agreed to by the supervising physi­cians and the responsible persons of HUMANA.

14. References

1. Benno Y, Sawada K, Mitsuoka T. The intestinal microflora of infants: Composition of the faecal flora in breast-fed and bottle-fed infants. Microbiol Immunol 1984;28:975-86
2. Gauhe AP, Gyorgy PA, Hoover JRE, et al. Bifidus factor. Preparations obtained from human milk. Arch Biochem 1954;48:214-24
3. Gibson GR, Roberfroid MB. Dietary modulation of the human colonic microbiota: introducing the concept of prebiotics. J Nutr 1995;125:1401-12
4. Fuller R. A review: probiotics in man and animals. J Appl Bacteriol 1989;66:365-78
5. Collins MD, Gibson GR. Probiotics, prebiotics, and synbiotics: approaches for modulating the microbial ecology of the gut. Am J Clin Nutr 1999;69(suppl):1052S-7S
6. Sako T, Matsumoto K, Tanaka R. Recent progress on research and applications of non-digestible galacto-oligosaccharides. International Dairy Journal 1999;9:69-80
7. Bouhnik Y, Flourié B, D’Agay-Abensour L, et al. Administration of Transgalacto-Oligosaccharides Increases Fecal Bifidobacteria and Modifies Colonic Fermentation Metabolism in Healthy Humans. J Nutr 1997;127:444-8
8. Teuri U, Korpela R. Galacto-oligosaccharides relieve constipation in elderly people. Ann Nutr Metab 1998;42:319-27
9. Kitajima H, Sumida Y, Tanaka R, et al. Early administration of Bifidobacterium breve to preterm infants: randomised controlled trial. Arch Dis Child Fetal Neonatal Ed 1997;76:F101-F107
10. Ha GY, Yang CH, Kim H, Chong Y. Case of Sepsis Caused by Bifidobacterium longum. J Clin Microbiol 1999;37:1227-8
11. Haschke F, Wang W, Ping G, et al. Clinical trials prove the safety and efficacy of the probiotic strain Bifidobacterium Bb12 in follow-up formula and growing-up milks. Monatsschr Kinderheilk 1998;146(suppl1):S26-S30
12. Saavedra JM Bauman NA, Oung I, et al. Feeding of Bifidobacterium bifidum and Streptococcus thermophilus to infants in hospital for prevention of diarrhoea and shedding of rotavirus. Lancet 1994;344:1046-49
13. Fukushima Y, Li ST, Hara H, et al. Effect of follow-up formula containing Bifidobacteria (NAN BF) on fecal flora and fecal metabolism in healthy children. Bioscience Microflora 1997;16:65-72
14. Crowther JS. Transport and Storage of faeces for bacteriological examination. J App Bacteriol 1971;34:477-83
15. de Vrese M, Schrezenmeir J. Pro- und Präbiotika - Stand der Diskussion. Ernährungs-Umschau 1998;45 (Sonderheft): S79-S89
16. de Vrese M, Stegelmann A, Richter B, Fenselau S, Laue C,Schrezenmeir J. Probiotics - Compensation for lactase insuffiency. Am J Clin Nutr (2001) 73:421S-429S
17. Scholz-Ahrens KE., Schaafsma G., van den Heuvel E.G.H.M., Schrezenmeir J. Effects of prebiotics on mineral metabolism. Am J Clin Nutr (2001) 73:459S-464S
18. SCHOLZ-AHRENS KE., VAN LOO J., SCHREZENMEIR J. Long term effect of oligofructose on bone trabecular structure in ovariectomized rats. Am J Clin Nutr (2001) 73:498S
19. SCHOLZ-AHRENS KE., VAN LOO J, SCHREZENMEIR J. Effect of oligofructose on bone mineralization in ovariectomized rats is affected by dietary calcium. Am J Clin Nutr (2001) 73:498S
20. Bennet R, Nord CE, Zetterstrom R. Transient colonization of the gut of newborn infants by orally administered bifidobacteria and lactobacilli. Acta Paediatr 1992 81: 784-7
21. Langhendries JP, Detry J, Van Hees J, Lamboray JM, Darimont J, Mozin MJ, Secretin MC, Senterre J. Effect of a fermented infant formula containing viable bifidobacteria on the fecal flora composition and pH of healthy full-term infants. J Pediatr Gastroenterol Nutr 1995;21:177-81
22. Millar MR, Bacon C, Smith SL, Walker V, Hall MA. Enteral feeding of premature infants with Lactobacillus GG. Arch Dis Child 1993;69:483-7
23. Phuapradit P, Varavithya W, Vathanophas K, Sangchai R, Podhipak A, Suthutvoravut U, Nopchinda S, Chantraruksa V, Haschke F. Reduction of rotavirus infection in children receiving bifidobacteria-supplemented formula. J Med Assoc Thai 1999;82 Suppl 1:S43-8
24. Stansbridge EM, Walker V, Hall MA, Smith SL, Millar MR, Bacon C, Chen S. Effects of feeding premature infants with Lactobacillus GG on gut fermentation. Arch Dis Child 1993 ;69:488-92
25. Walter J, Hertel C, Tannock GW, Lis CM, Munro K, Hammes WP (2001) Detection of Lactobacillus, Pediococcus, Leuconostoc, and Weissella Species in Human Feces by Using Group-Specific PCR Primers and Denaturing Gradient Gel Electrophoresis. Appl Environ Microbiol 67: 2578-2585
26. Ott SJ, Musfeldt M, Ullmann U, Hampe J, Schreiber S (2004) Quantification of intestinal bacterial populations by real-time PCR using a universal primer set and MGB probes: a global approach to the enteric flora. J Clin Microbiol 42: 2566-2572
27. Langendijk PS, Schut F, Jansen GJ, Raangs GC, Kamphuis GR et al. (1995) Quantitative

fluorescence in situ hybridization of Bifidobacterium spp. with genus-specific 16S rRNA- targeted probes and its application in fecal samples. Appl Environ Microbiol 61: 3069-3075

1. Bernhard AE, Field KG (2000) Identification of nonpoint sources of fecal pollution in coastal waters by using host-specific 16S ribosomal DNA genetic markers from fecal anaerobes. Appl Environ Microbiol 66: 1587-1594
