# Supplementary material for: Prebiotics Do Not Influence the Severity of Atopic Dermatitis in Infants: A Randomised Controlled Trial
Source: PLoS One. 2015 Nov 16;10(11):e0142897. doi: 10.1371/journal.pone.0142897 (PMC4646669; doi:10.1371/journal.pone.0142897)
Supplement: S2 File — (DOC) [file pone.0142897.s003.doc]

**Dvojiteslepa randomisovana studie o vlivu prebiotik na incidenci atopickych komplikaci u kojencu - synopse**

## Projekt no.: 1.090

Datum:15.1.2007

**Odpovedny lekar:**

Prim MUDr.J .Bozensky, Vitkovicka nemocnice, Zaluzanskeho Ostrava

**Sponsor:**

HUMANA GmbH

Bielefelder Str. 66

D-32051 Herford / Germany

Tel: ++49-5221-181-0

Fax: ++49-5221-181-0

**1.** PROTOKOL

**CILE:**

Vyhodnotit vliv pridavku probiotika na incidenci atopie o kojencu s pozitivni rodinnou alergickou anamnezou

**DESIGN:**

Dvojte slepa randomisovana kontrolovana prospektivni studie

**POCET PROBANDU:**

60 deti ve skupine, dve skupiny,

**SELEKCNI CHARAKTERISTIKY:**

Kojenci s pozitivni rodinnou alergickou anamnezou (narozeni mezi 37. and 42.tydnem gestace), nehypotroficti, po 6 tydnech veku umela vyziva jediny zdroj vyzivy

**SKUPINY:**

2 skupiny s ruznymi HA pripravky: (standard and obohacene prebiotiky)

**Vyziva:**

Ad libitum

**Hlavni merene parametry:**

Analyza SCORAD, vyhodnoceni udaju o incidenci place, regurgitace a o kontzistenci stolice

**Parametry bezpecnosti:**

Normalni vyvoj podle rustovych tabulek.

**POSTUP:**

**Deti vysetri lekar v case 0, 3, a 6 mesicu Ostatni parametry budou zznamenavany rodici**

**STATISTICKA ANALYZA:**

Pomoci Mann-Whitney a chi square analyza frekvenci

2. Primarni hypoteza

Prepokladam,e ze incidenci atopii u deti krmenych vyzivou s pridavkem GOS bude minimalne o 30% nizsi, v ostatnich parametrech se skupiny nebudou lisit..

3.Vyhodnocovane parametry

**Primarni:**

SCORAD hodnota

**Sekundarni:**

Hmotnost, delka, objem vypite formule).

Frekvence a konzistence stolice

Plenkova dermatitida

Prujmy, horecky, krece, placi a regurgitace

**Bezpecnostni:**

Normalni vyvoj podle rustovych krivek

5. Design

Dvojite slepa randomisovana prospektivni studie .

.

**6. Trvani**

12 mesicu, predpoklad dokonceni studie do podzimu 2008

**Nezadouci ucinky**

Veskere negativni ucinky budou hlaseny koordinujicimu lekari , na zaklade jeho posouzeni potom budou podniknuty dalsi kroky

Sponzor uzavrel pojisteni u spolecnosti Gothaer Versicherung, kryjici veskera rizika studie**.**
